# Severe Dengue Fever with Haemolytic Anaemia—A Case Study

**DOI:** 10.3390/tropicalmed1010006

**Published:** 2016-10-08

**Authors:** Mra Aye, Jason Cabot, Lee Wei Kiat William

**Affiliations:** 1 Department of Medicine, Melaka Manipal Medical College, Melaka 75150, Malaysia; 2 Consultant oncohematologist, 633 Gov. Carlos Canaco Rd., B5, Tamung, Guam 96913, USA; beaunestay@gmail.com; 3 Melaka General Hospital, Melaka 75150, Malaysia; weikiatlee@hotmail.com

**Keywords:** severe dengue fever, haemolytic anaemia

## Abstract

Dengue fever, the most common arthropod-borne viral infection in South East Asia, is increasing in prevalence due partially to increased awareness and better diagnostic methods. While haematologic complications, such as cytopeniae and bleeding, may occur in severe dengue infection due to a variety of aetiologies, reports of haemolytic anaemia in dengue fever are scant. We report a case of severe dengue fever with haemolytic anaemia following the critical phase of infection.

## 1. Case History

A 22-year-old male was admitted with a three-day history of high-grade continuous fever with chills, vomiting and loose stools. He denied abdominal pain, myalgia, retro-orbital pain, back pain or other systemic symptoms. He had no previous history of dengue fever. Three family members, (father, mother and grandfather) had simultaneously similar, but milder symptoms. As tests for non-structural protein (NS1) (SD Bioline, Standard Diagnostics Inc., Gyeonggi-do, Korea) and IgM against dengue virus (Panbio, Alere, Brisbane) were positive, he was diagnosed with dengue fever. On day two post-admission (day five of illness, the critical phase) he developed haematoma formation at venipuncture sites and unprovoked gingival bleeding. Platelets dropped from 35 × 10^9^ to 14 × 10^9^/L and haematocrit was stable at 45% with stable haemodynamic status (Table 1). On day three post-admission he developed tachycardia, mild abdominal pain and tenderness, and falling blood pressure. He was seen to have compensated metabolic acidosis and prolonged activated partial thromboplastin time (APTT) (Table 2). Abdominal ultrasound revealed bilateral basal pleural effusions, ascites and hepatitis with alanine aminotransferase (ALT) values rising from 112 U/L to 212 U/L. He was haemodynamically stable. Intravenous isotonic crystalline fluid (3 mL/kg/h) was administered for the first 4 hours and blood pressure, clinical status and full blood count were monitored. The fluid therapy was continued in the same dose or decreased to 1–2 mL/Kg/h accordingly to blood pressure, clinical status, and haematocrit until day 3 post-admission. On day four post-admission (day seven of illness) he recovered from the critical phase and intravenous fluid therapy was stopped. However, on day five post-admission (day 8 of illness) there was sudden drop of haemoglobin from 15 to 10 g/dL, with continued drop to 8.5 g/dL on the subsequent day, and elevated white blood cell count (13 × 10^9^/L) with platelet count having risen to 112 × 10^9^ /L (Table 1).

Stool for occult blood was negative and there were no bleeding manifestations. Liver, spleen and lymph nodes were not enlarged. Other system examinations were normal except for slight jaundice, and a haemolysis work-up was performed. The peripheral blood film was compatible with haemolysis (Table 1). Indirect bilirubin was higher than direct bilirubin on day 1 (61.5 µmol and 9.6 µmol, respectively), AST (aspartate aminotransferase) was higher than ALT (alanine aminotransferase) on day 5 (385 U/L and 202 U/L, respectively), LDH (lactate dehydrogenase) was very high (2013–1708 U/L), indirect Coombs’ test was positive and reticulocyte count was grossly elevated (10.3%) (Table 1). Tests for glucose-6-phosphate deficiency, antinuclear antibody, Epstein-Barr virus, human immunodeficiency virus, and hepatitis A, B and C antibodies, were negative. As the anaemia was relatively asymptomatic, conservative treatment was continued. Haemoglobin rose to 12 g/dL in next three days. He was discharged with normal haemoglobin, WBC and platelets. Anti-dengue IgM and IgG antibodies in blood drawn two weeks after discharge subsequently were positive (Table 2).

## 2. Discussion

Our case of severe dengue fever with impending dengue shock syndrome [1], developed hemolysis on day 9 of illness, as manifested by sudden drop of haemoglobin, reticulocytosis, positive indirect Coombs’s test, and no blood loss, with rising platelet count (after severe depression) and normal coagulation studies. Haemolytic anaemia in dengue fever is considered rare, and has been described in case reports in Sri Lanka [2], in India as cold agglutinin-induced haemolytic anaemia in a dengue patient [3], and in a British traveller [4].

Although clinically detectable haemolysis occurred on day 6 of admission (day 9 of illness) in our patient, higher indirect than direct bilirubin was noted since the first day of admission, together with high AST and LDH (Table 1), suggesting asymptomatic haemolysis was present since admission. High LDH cannot be used as sole indicator of haemolysis in DHF since significant elevations of LDH, AST and CK are usual findings in severe dengue cases due to ischemic tissue injury [5,6]. Higher indirect than direct bilirubin in dengue fever was reported in only 15% of cases in India [7], suggesting that dengue fever cases with this finding may indicate some haemolysis, as in our patient.

Our case developed a positive indirect Coombs’ test (Table 1) differing from other cases that were Coombs’ negative. Dengue virus may alter antigens on red blood cell membranes and cross-react with antibody directed against the virus; there may be different immune-related mechanisms by which antibodies are directed at antigens developed on RBC membranes by various dengue virus serotypes. 

The pathophysiology of thrombocytopenia and leucopenia in dengue fever is poorly understood, but hypotheses such as depression of bone marrow, direct invasion of virus in monocytes and platelets, and increased consumption and destruction, have been proposed, with red blood cells possibly being relatively resistant to viral invasion [8].

Our patient was noted to have monocytosis until day 12. Monocytosis is reported to be the most, or second most, common haematologic manifestation in dengue fever [9,10]. Our patient did not have leucopenia. In the study by Malathesha et al. [9], leucopenia was seen in 27.6% of cases, lymphocytosis (>45%) in 66%, monocytosis (>10%) in 84.6%, basophilia (>2%) in 52.9%, and 44.4% had platelet counts below 50 × 10^9^/L. 

Dengue fever with early alterations of biochemical markers such as high LDH, CK, and AST, and lower levels of albumin, total cholesterol, and triglycerides can predict severe dengue disease [11]. Additionally, rise of LDH, CK and AST (Table 1) together are not only predictors of severe dengue, but may also be biochemical features of rhabdomyolysis [12,13]. In our case, there were no clinical symptoms or signs to suggest rhabdomyolysis, the renal profile was normal, and the clinical picture was dominated by marked pallor, jaundice and reticulocytosis. 

With the sudden anaemia and elevated APTT, disseminated intravascular coagulopathy was considered, but ultimately thought unlikely as the peripheral blood film lacked microangiopathic features, and prothrombin time (PT) and fibrinogen were normal. 

Our patient had acute abdominal pain on day three of admission and reports of acute abdominal pain in dengue fever mimicking acute abdomen with acute acalculous cholecystitis, acute hepatitis, acute pancreatitis and acute enteritis, are described [14,15,16]. Our patient was thought to have hepatitis because of ultrasound examination, and higher ALT than AST levels. Although dengue fever is characterized by either single or multiple cytopeniae (thrombocytopenia and/or leucopenia), similar peripheral blood pictures can be seen in bacterial sepsis and other viraemias. Prolongation of APTT may help differentiate dengue from surgical causes of abdominal pain especially acute appendicitis [14], and the abdominal pain of dengue is commonly relieved by fluid replacement. Prolongation of APTT with normal prothrombin time, a characteristic feature of severe dengue [17,18], is thought to be due to imbalance between intrinsic and extrinsic coagulation systems [17].

## 3. Conclusions

We present a case of dengue fever with a short duration of a brisk haemolytic anaemia with a positive indirect Coombs’ test, manifesting in the second week of illness after recovery from the critical period (and impending dengue shock syndrome). There had been higher indirect than direct bilirubin since first day of admission (day three of illness), indicating there may have been chronic ongoing haemolysis preceding the acute, brisk episode. Our case implies that immune haemolytic anaemia might be a potential clinical feature of severe dengue. It alerts clinicians to consider haemolysis as a cause of anaemia other than blood loss due to gastrointestinal bleeding, in dengue. It also implies that dengue virus infection might be included in the list of the infective causes of immune haemolytic anaemia in addition to *Mycoplasma pneumoniae* and others.

## Figures and Tables

**Table 1:** Relevant haematology and biochemistry investigations, by day of admission.

Laboratory Investigations	Day 1 *	Day 3	Day 5	Day 7	Day 9	Day 15	Normal Range
Haemoglobin	15.0	15.5	10.2	8.4	9.1	11.0	13–17 g/dL
Red blood cell count	5.15	5.14	3.32	2.9	3.03	3.70	1.50–5.50 × 10^12^/L
Haematocrit	45.3	46	26.7	25.6	27	33.9	10%–50%
White blood cell count	3.1	6.0	11.4	14.1	8.9	7.5	4.00–10.0 × 10^9^/L
Platelets	35	14	29	127	136	259	150–410 ×10^9^/L
Monocytes %	12.2	17.3	15.4	22.8	18	10.1	2%–10%
Reticulocyte count				10.8%			
Indirect Coomb’s test				Positive			
Serum bilirubin							
• Total	71.4	65.6	51.9	59.9	48.9	48.7	<18.7 µmol/L
• Direct	9.6	30.1	21.5	17.2	14.3		<5.7 µmol/L
• Indirect	61.5	35.5	30.2	42.7	34.6		2.00–3.00 µmol/L
Serum enzymes							
• AST	87		385	187	131		1–40 U/L
• ALT	112	109	202	156	126	80	1–41 U/L
• CK	487		1331	465	298		<190 U/L
• LDH		1090	2013		1063		240–480 U/L
Urea	1.9	2.31	3.2	1.9	2.5		1.7–8.3 mmol/L
APTT			56.8				27.7–41 s
Serum lactate			2.2				0.2–2.0 mmol/L

* Day of admission, AST= aspartate aminotransferase, ALT = alanine aminotransferase, CK = creatinine kinase, LDH = lactate dehydrogenase, APTT = activated partial thromboplastin time.

**Table 2:** Other investigations, by day of admission.

Investigation	Day 1 *	Day 3	Day 5	Day 29
Dengue NS1 antigen	Positive			
Dengue IgM			Positive	Positive
Dengue IgG				Positive
Ultrasound of hepatobiliary system		Hepatitis, ascites, pleural effusion		

* Day of admission.
